# *cis*-Dichloridobis[tris­(2-methyl­phen­oxy)phosphane-κ*P*]palladium(II)

**DOI:** 10.1107/S160053681200342X

**Published:** 2012-02-10

**Authors:** Izabela Błaszczyk, Anna M. Trzeciak, Andrzej Gniewek

**Affiliations:** a Faculty of Chemistry, University of Wrocław, 14 F. Joliot-Curie, 50-383 Wrocław, Poland

## Abstract

In the title compound, [PdCl_2_(C_21_H_21_O_3_P)_2_], the Pd atom adopts a slightly distorted square-planar coordination geometry, with pairs of the equivalent ligands in *cis* positions. Adjacent mol­ecules are linked by weak C—H⋯Cl hydrogen bonds. The crystal structure is additionally stabilized by π–π stacking inter­actions between the aromatic rings [shortest centroid–centroid distance = 3.758 (4) Å].

## Related literature

The structure of the title compound was determined as part of a larger study on palladium(II) complexes with triphenyl­phosphito ligands. For related structures and further discussion, see: Błaszczyk *et al.* (2009 ▶); Sabounchei *et al.* (2000 ▶); Trzeciak *et al.* (2001 ▶). For the Sonogashira reaction, see: Sonogashira *et al.* (1975 ▶). For bond lengths in coordination complexes, see: Orpen *et al.* (1989 ▶). For hydrogen-bond inter­actions, see: Aullón *et al.* (1998 ▶); Desiraju & Steiner (1999 ▶); and for π–π stacking contacts, see: McGaughey *et al.* (1998 ▶). For details of the temperature control applied during data collection, see: Cosier & Glazer (1986 ▶); and for specifications of analytical numeric absorption correction, see: Clark & Reid (1995 ▶).
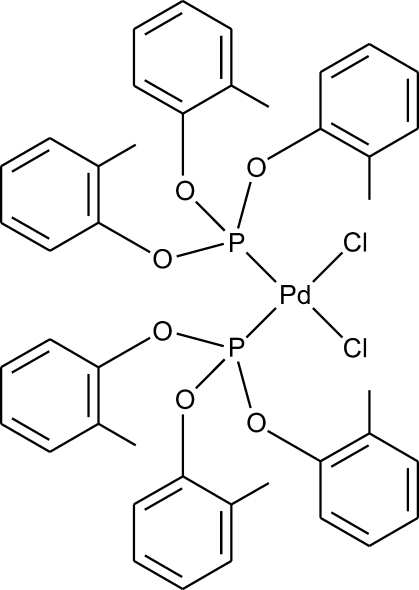


## Experimental

### 

#### Crystal data

[PdCl_2_(C_21_H_21_O_3_P)_2_]
*M* *_r_* = 882.00
Triclinic, 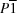

*a* = 9.575 (3) Å
*b* = 12.248 (4) Å
*c* = 17.814 (5) Å
α = 106.12 (3)°
β = 90.42 (3)°
γ = 98.74 (3)°
*V* = 1981.0 (11) Å^3^
*Z* = 2
Mo *K*α radiation
μ = 0.73 mm^−1^
*T* = 100 K
0.40 × 0.32 × 0.16 mm

#### Data collection

Kuma KM-4 CCD diffractometer
Absorption correction: analytical (*CrysAlis RED*; Oxford Diffraction, 2010 ▶) *T*_min_ = 0.848, *T*_max_ = 0.902
26365 measured reflections
9077 independent reflections
7974 reflections with *I* > 2σ(*I*)
*R*_int_ = 0.025

#### Refinement

*R*[*F* ^2^ > 2σ(*F* ^2^)] = 0.034
*wR*(*F* ^2^) = 0.093
*S* = 1.08
9077 reflections
484 parameters
H-atom parameters constrained
Δρ_max_ = 1.61 e Å^−3^
Δρ_min_ = −0.67 e Å^−3^

### 

Data collection: *CrysAlis CCD* (Oxford Diffraction, 2010 ▶); cell refinement: *CrysAlis RED* (Oxford Diffraction, 2010 ▶); data reduction: *CrysAlis RED*; program(s) used to solve structure: *SHELXS97* (Sheldrick, 2008 ▶); program(s) used to refine structure: *SHELXL97* (Sheldrick, 2008 ▶); molecular graphics: *ORTEP-3* (Farrugia, 1997 ▶); software used to prepare material for publication: *SHELXL97*.

## Supplementary Material

Crystal structure: contains datablock(s) global, I. DOI: 10.1107/S160053681200342X/bt5800sup1.cif

Structure factors: contains datablock(s) I. DOI: 10.1107/S160053681200342X/bt5800Isup2.hkl

Additional supplementary materials:  crystallographic information; 3D view; checkCIF report

## Figures and Tables

**Table:** 

Pd—P1	2.2254 (9)
Pd—P2	2.2296 (9)
Pd—Cl1	2.3375 (9)
Pd—Cl2	2.3164 (9)

**Table:** 

Cl1—Pd—Cl2	90.59 (2)
P1—Pd—P2	94.07 (2)
P1—Pd—Cl1	90.35 (2)
P2—Pd—Cl2	85.09 (2)
P2—Pd—Cl1	175.50 (2)

**Table 2:** Hydrogen-bond geometry (Å, °)

*D*—H⋯*A*	*D*—H	H⋯*A*	*D*⋯*A*	*D*—H⋯*A*
C17—H17*A*⋯Cl2^i^	0.98	2.72	3.521 (3)	139
C45—H45⋯Cl1^ii^	0.95	2.91	3.680 (3)	139

Symmetry codes: (i) 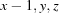; (ii) 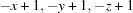.

**Table 3:** Inter­molecular π–π inter­actions (Å, °) *Cg*1 denotes the centroid of ring C11–C16; *Cg*2 of ring C41–C46. *Cg*⋯*Cg* is the distance between ring centroids. The inter­planar distance is the perpendicular distance of *CgI* from the ring *J* plane. The offset is the lateral displacement of ring *I* relative to ring *J*. The planes of the *I* and *J* rings are parallel.

*CgI*	*CgJ*	*Cg*⋯*Cg*	Inter­planar distance	Offset
1	1^iii^	3.758 (4)	3.409 (4)	1.582 (4)

Symmetry codes: (iii) −*x*, −*y* + 1, −*z* + 1; (iv) −*x* + 1, −*y*, −*z* + 1.

**Fig. 1.**
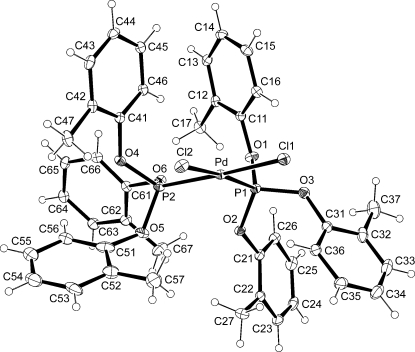
The molecular structure and atom numbering scheme of the title compound. Displacement ellipsoids are drawn at the 30% probability level and H atoms are shown as small spheres of arbitrary radii.

## supplementary crystallographic information

### Comment

Palladium complexes with phosphito ligands are frequently used as catalyst precursors in carbon-carbon bond-forming reactions. The Sonogashira reaction has attracted a lot of attention as an efficient way to produce phenylated alkines (Sonogashira *et al.*, 1975). In this paper we report crystallization of a palladium(II) complex with tritolylphosphito ligands, the title compound, which has recently proved its high catalytic activity in a copper-free Sonogashira reaction with iodobenzene and phenylacetylene as substrates and imidazolium ionic liquids as the reaction medium (Błaszczyk *et al.*, 2009).

The Pd atom of the title compound is four-coordinated in a square-planar geometry (Fig. 1). The molecule adopts the *cis* configuration in the solid state. The angles between adjacent ligands deviate only slightly from the expected value of 90° (Table 1). The Pd—Cl1 and Pd—Cl2 bond distances are within a range typical for palladium complexes: 2.298–2.354Å (Orpen *et al.*, 1989). The measured Pd—P bond lengths 2.22–2.24Å are also commonly observed in such a kind of complexes (Sabounchei *et al.*, 2000; Trzeciak *et al.* 2001).

In the crystal structure, the molecules of the title complex are linked by a few weak hydrogen interactions of the C—H···Cl type (Desiraju & Steiner, 1999). The C17 and C45 atoms act as hydrogen-bond donors, *via* H17A and H45, to the Cl^i^ or Cl^ii^ atom [symmetry codes: (i) *x* - 1, *y*, *z*; (ii) -*x* + 1, -*y* + 1, -*z* + 1], respectively, as an acceptor (Table 2). The observed C—H···Cl distances are similar to the values of the N—H···Cl hydrogen bonds identified for Cl bonded to a transition metal (Aullón *et al.*, 1998).

Additionally, the C11—C16 and C41—C46 aromatic rings are engaged in π-π stacking contacts, which further assist in the stabilization of the crystal structure (Table 3). Even though the distance of the centroids and the offset of the interacting rings may first appear to be somewhat large, it is however well known that energetically favorable non-bonded aromatic interactions are generally observed at such phenyl ring centroid separation distances (McGaughey *et al.*, 1998).

### Experimental

The title compound was prepared according to the previously reported procedure (Błaszczyk *et al.*, 2009): tris(2-methylphenyl)phosphite (0.96 ml, 3.0 mmol) was slowly added to the solution of PdCl_2_(cyclooctadiene) (0.144 g, 0.6 mmol) in benzene (5 ml). A change of color from yellow to pale yellow was observed. The solution was stirred at room temperature for 45 minutes. The solvent was evaporated *in vacuo*. The white product was recrystallized from a mixture of benzene and diethyl ether. Yield: 0.25 g, 48%. Analysis calculated: C 57.19, H 4.80; found: C 57.20, H 4.71%. IR (KBr, cm^-1^): ν(=C—H) 3066, 3033, 2961, 2928, ν(C=C) 1584, 1491, 1460, ν(P—O—C) 1226, 1162, 1110, 1044, ν(C—H) 952, 803, 762, 736, 609. ^1^H NMR (CDCl_3_): δ 1.00 (6*H*, t, ^3^J = 4.5 Hz), 6.14–7.03 (m, Ph), 2.07 (3*H*, s, CH_3_). ^31^P NMR (CDCl_3_): δ 81.27.

### Refinement

All H atoms were positioned geometrically and refined using a riding model with aromatic C—H = 0.95Å and *U*_iso_(H) = 1.2*U*_eq_(C). The methyl groups were refined with C—H = 0.98Å and *U*_iso_(H) = 1.5*U*_eq_(C). The highest residual peak and the deepest hole in the final difference map are located 0.83 and 0.77Å from the C51 and Pd atom, respectively.

### Crystal data

**Table:**

[PdCl_2_(C_21_H_21_O_3_P)_2_]	*Z* = 2
*M**_r_* = 882.00	*F*(000) = 904
Triclinic, *P*1	*D*_x_ = 1.479 Mg m^−^^3^
Hall symbol: -P 1	Mo *K*α radiation, λ = 0.71073 Å
*a* = 9.575 (3) Å	Cell parameters from 18240 reflections
*b* = 12.248 (4) Å	θ = 5.0–27.5°
*c* = 17.814 (5) Å	µ = 0.73 mm^−^^1^
α = 106.12 (3)°	*T* = 100 K
β = 90.42 (3)°	Plate, colorless
γ = 98.74 (3)°	0.40 × 0.32 × 0.16 mm
*V* = 1981.0 (11) Å^3^	

### Data collection

**Table:**

Kuma KM-4 CCD diffractometer	9077 independent reflections
Radiation source: fine-focus sealed tube	7974 reflections with *I* > 2σ(*I*)
Graphite monochromator	*R*_int_ = 0.025
ω scans	θ_max_ = 27.5°, θ_min_ = 5.0°
Absorption correction: analytical (*CrysAlis RED*; Oxford Diffraction, 2010)	*h* = −12→12
*T*_min_ = 0.848, *T*_max_ = 0.902	*k* = −15→15
26365 measured reflections	*l* = −16→23

### Refinement

**Table:**

Refinement on *F*^2^	Primary atom site location: heavy-atom method
Least-squares matrix: full	Secondary atom site location: difference Fourier map
*R*[*F*^2^ > 2σ(*F*^2^)] = 0.034	Hydrogen site location: inferred from neighbouring sites
*wR*(*F*^2^) = 0.093	H-atom parameters constrained
*S* = 1.08	*w* = 1/[σ^2^(*F*_o_^2^) + (0.0544*P*)^2^ + 1.3532*P*] where *P* = (*F*_o_^2^ + 2*F*_c_^2^)/3
9077 reflections	(Δ/σ)_max_ = 0.001
484 parameters	Δρ_max_ = 1.61 e Å^−^^3^
0 restraints	Δρ_min_ = −0.67 e Å^−^^3^

### Special details

**Table:**

Experimental. The crystal was placed in the cold stream of an open-flow nitrogen cryostat (Cosier & Glazer, 1986) operating at 100 K. Analytical numeric absorption correction was carried out with *CrysAlis RED* (Oxford Diffraction, 2010) using a multifaceted crystal model (Clark & Reid, 1995).
Geometry. All e.s.d.'s (except the e.s.d. in the dihedral angle between two l.s. planes) are estimated using the full covariance matrix. The cell e.s.d.'s are taken into account individually in the estimation of e.s.d.'s in distances, angles and torsion angles; correlations between e.s.d.'s in cell parameters are only used when they are defined by crystal symmetry. An approximate (isotropic) treatment of cell e.s.d.'s is used for estimating e.s.d.'s involving l.s. planes.
Refinement. Refinement of *F*^2^ against ALL reflections. The weighted *R*-factor *wR* and goodness of fit *S* are based on *F*^2^, conventional *R*-factors *R* are based on *F*, with *F* set to zero for negative *F*^2^. The threshold expression of *F*^2^ > 2σ(*F*^2^) is used only for calculating *R*-factors(gt) *etc*. and is not relevant to the choice of reflections for refinement. *R*-factors based on *F*^2^ are statistically about twice as large as those based on *F* and *R*- factors based on ALL data will be even larger.

### Fractional atomic coordinates and isotropic or equivalent isotropic displacement parameters (Å^2^)

**Table:**

	*x*	*y*	*z*	*U*_iso_*/*U*_eq_	
Pd	0.400484 (15)	0.294943 (13)	0.282022 (9)	0.01762 (6)	
Cl1	0.51620 (5)	0.47374 (5)	0.27470 (3)	0.02515 (12)	
Cl2	0.61585 (5)	0.23734 (5)	0.29736 (3)	0.02538 (12)	
P1	0.19314 (5)	0.34091 (5)	0.25564 (3)	0.01771 (11)	
P2	0.30605 (5)	0.12238 (5)	0.29288 (3)	0.01802 (11)	
O1	0.09044 (15)	0.36632 (14)	0.32508 (9)	0.0234 (3)	
O2	0.10765 (15)	0.23789 (13)	0.18878 (9)	0.0204 (3)	
O3	0.18784 (16)	0.45217 (13)	0.22691 (9)	0.0233 (3)	
O4	0.38304 (15)	0.07301 (12)	0.35210 (9)	0.0202 (3)	
O5	0.29919 (18)	0.01967 (14)	0.21472 (9)	0.0272 (3)	
O6	0.14500 (15)	0.11591 (12)	0.31591 (9)	0.0190 (3)	
C11	0.1329 (2)	0.39945 (17)	0.40527 (12)	0.0175 (4)	
C12	0.0448 (2)	0.35074 (17)	0.45303 (13)	0.0184 (4)	
C13	0.0815 (2)	0.39022 (19)	0.53348 (13)	0.0217 (4)	
H13	0.0228	0.3608	0.5684	0.026*	
C14	0.2007 (2)	0.4708 (2)	0.56355 (13)	0.0244 (4)	
H14	0.2233	0.4951	0.6183	0.029*	
C15	0.2869 (2)	0.51586 (19)	0.51349 (14)	0.0243 (4)	
H15	0.3695	0.5704	0.5338	0.029*	
C16	0.2523 (2)	0.48113 (18)	0.43391 (13)	0.0211 (4)	
H16	0.3095	0.5128	0.3993	0.025*	
C17	−0.0846 (2)	0.26301 (19)	0.42069 (14)	0.0243 (4)	
H17A	−0.1284	0.2818	0.3769	0.037*	
H17B	−0.1523	0.2637	0.4618	0.037*	
H17C	−0.0575	0.1864	0.4023	0.037*	
C21	−0.0088 (2)	0.24092 (18)	0.14012 (13)	0.0204 (4)	
C22	−0.0023 (2)	0.18617 (18)	0.06101 (13)	0.0234 (4)	
C23	−0.1180 (3)	0.1846 (2)	0.01254 (14)	0.0287 (5)	
H23	−0.1174	0.1483	−0.0420	0.034*	
C24	−0.2342 (3)	0.2349 (2)	0.04228 (15)	0.0303 (5)	
H24	−0.3115	0.2334	0.0081	0.036*	
C25	−0.2371 (2)	0.2871 (2)	0.12163 (15)	0.0294 (5)	
H25	−0.3169	0.3208	0.1420	0.035*	
C26	−0.1233 (2)	0.2904 (2)	0.17182 (14)	0.0244 (4)	
H26	−0.1244	0.3257	0.2265	0.029*	
C27	0.1245 (3)	0.1318 (2)	0.03005 (14)	0.0308 (5)	
H27A	0.2052	0.1923	0.0317	0.046*	
H27B	0.1027	0.0843	−0.0241	0.046*	
H27C	0.1480	0.0836	0.0624	0.046*	
C31	0.2144 (2)	0.46312 (19)	0.15151 (13)	0.0233 (4)	
C32	0.1324 (3)	0.5317 (2)	0.12557 (17)	0.0319 (5)	
C33	0.1604 (3)	0.5475 (2)	0.05248 (18)	0.0418 (7)	
H33	0.1072	0.5941	0.0330	0.050*	
C34	0.2627 (3)	0.4978 (3)	0.00751 (17)	0.0426 (7)	
H34	0.2789	0.5101	−0.0423	0.051*	
C35	0.3421 (3)	0.4299 (2)	0.03485 (15)	0.0336 (5)	
H35	0.4128	0.3955	0.0039	0.040*	
C36	0.3180 (2)	0.41223 (19)	0.10773 (13)	0.0253 (4)	
H36	0.3718	0.3659	0.1271	0.030*	
C37	0.0227 (3)	0.5874 (3)	0.1752 (2)	0.0452 (7)	
H37A	−0.0514	0.5278	0.1834	0.068*	
H37B	−0.0192	0.6360	0.1488	0.068*	
H37C	0.0669	0.6349	0.2259	0.068*	
C41	0.4445 (2)	0.13559 (17)	0.42680 (12)	0.0181 (4)	
C42	0.5674 (2)	0.10157 (18)	0.44879 (13)	0.0204 (4)	
C43	0.6253 (2)	0.1583 (2)	0.52377 (14)	0.0254 (5)	
H43	0.7077	0.1363	0.5415	0.030*	
C44	0.5661 (2)	0.2460 (2)	0.57344 (14)	0.0254 (5)	
H44	0.6090	0.2840	0.6241	0.030*	
C45	0.4445 (2)	0.27822 (18)	0.54932 (13)	0.0225 (4)	
H45	0.4042	0.3385	0.5832	0.027*	
C46	0.3821 (2)	0.22198 (18)	0.47544 (13)	0.0195 (4)	
H46	0.2979	0.2424	0.4585	0.023*	
C47	0.6355 (2)	0.0108 (2)	0.39316 (16)	0.0281 (5)	
H47A	0.6711	0.0393	0.3495	0.042*	
H47B	0.5655	−0.0590	0.3730	0.042*	
H47C	0.7142	−0.0069	0.4207	0.042*	
C51	0.3775 (3)	−0.0766 (3)	0.19830 (15)	0.0367 (6)	
C52	0.4797 (3)	−0.0809 (2)	0.14459 (16)	0.0364 (6)	
C53	0.5453 (3)	−0.1784 (3)	0.12622 (18)	0.0479 (8)	
H53	0.6163	−0.1847	0.0890	0.058*	
C54	0.5094 (3)	−0.2663 (3)	0.16095 (18)	0.0462 (7)	
H54	0.5561	−0.3317	0.1476	0.055*	
C55	0.4059 (4)	−0.2590 (3)	0.21493 (18)	0.0504 (8)	
H55	0.3838	−0.3188	0.2393	0.061*	
C56	0.3332 (4)	−0.1656 (2)	0.23427 (16)	0.0426 (7)	
H56	0.2586	−0.1615	0.2693	0.051*	
C57	0.5158 (4)	0.0136 (3)	0.1097 (2)	0.0504 (8)	
H57A	0.4312	0.0244	0.0830	0.076*	
H57B	0.5885	−0.0045	0.0718	0.076*	
H57C	0.5519	0.0845	0.1507	0.076*	
C61	0.0641 (2)	0.01390 (17)	0.32605 (12)	0.0186 (4)	
C62	−0.0410 (2)	−0.04696 (19)	0.26908 (13)	0.0236 (4)	
C63	−0.1217 (2)	−0.1447 (2)	0.28173 (14)	0.0259 (5)	
H63	−0.1938	−0.1889	0.2438	0.031*	
C64	−0.0992 (2)	−0.17885 (19)	0.34812 (14)	0.0253 (5)	
H64	−0.1544	−0.2464	0.3550	0.030*	
C65	0.0040 (2)	−0.1142 (2)	0.40449 (14)	0.0241 (4)	
H65	0.0182	−0.1365	0.4506	0.029*	
C66	0.0869 (2)	−0.01668 (19)	0.39380 (13)	0.0210 (4)	
H66	0.1579	0.0281	0.4322	0.025*	
C67	−0.0716 (3)	−0.0070 (3)	0.19915 (16)	0.0396 (7)	
H67A	−0.1080	0.0661	0.2163	0.059*	
H67B	−0.1423	−0.0649	0.1637	0.059*	
H67C	0.0157	0.0040	0.1718	0.059*	

### Atomic displacement parameters (Å^2^)

**Table:**

	*U*^11^	*U*^22^	*U*^33^	*U*^12^	*U*^13^	*U*^23^
Pd	0.01242 (8)	0.02194 (9)	0.01643 (9)	−0.00119 (6)	0.00002 (6)	0.00415 (6)
Cl1	0.0209 (2)	0.0251 (3)	0.0260 (3)	−0.00535 (19)	−0.0009 (2)	0.0066 (2)
Cl2	0.0125 (2)	0.0378 (3)	0.0266 (3)	0.0014 (2)	0.00082 (19)	0.0118 (2)
P1	0.0145 (2)	0.0209 (3)	0.0166 (2)	−0.00023 (19)	−0.00008 (19)	0.0050 (2)
P2	0.0143 (2)	0.0201 (3)	0.0169 (2)	0.00000 (19)	0.00052 (19)	0.0020 (2)
O1	0.0148 (7)	0.0357 (9)	0.0186 (7)	0.0030 (6)	0.0003 (6)	0.0065 (6)
O2	0.0190 (7)	0.0221 (7)	0.0189 (7)	0.0002 (6)	−0.0041 (6)	0.0060 (6)
O3	0.0227 (7)	0.0220 (7)	0.0245 (8)	0.0017 (6)	0.0023 (6)	0.0063 (6)
O4	0.0164 (7)	0.0181 (7)	0.0234 (8)	0.0030 (5)	−0.0008 (6)	0.0012 (6)
O5	0.0297 (8)	0.0252 (8)	0.0209 (8)	0.0006 (7)	0.0008 (6)	−0.0010 (6)
O6	0.0139 (6)	0.0211 (7)	0.0217 (7)	−0.0004 (5)	0.0000 (5)	0.0072 (6)
C11	0.0154 (9)	0.0197 (9)	0.0176 (9)	0.0059 (7)	0.0000 (7)	0.0039 (8)
C12	0.0132 (9)	0.0182 (9)	0.0254 (10)	0.0056 (7)	0.0029 (8)	0.0072 (8)
C13	0.0212 (10)	0.0254 (10)	0.0223 (10)	0.0096 (8)	0.0051 (8)	0.0096 (9)
C14	0.0254 (11)	0.0269 (11)	0.0198 (10)	0.0100 (9)	−0.0011 (8)	0.0016 (9)
C15	0.0186 (10)	0.0218 (10)	0.0281 (11)	0.0022 (8)	−0.0023 (8)	0.0005 (9)
C16	0.0168 (9)	0.0198 (10)	0.0257 (11)	0.0031 (8)	0.0026 (8)	0.0048 (8)
C17	0.0154 (9)	0.0253 (11)	0.0312 (12)	0.0008 (8)	0.0035 (8)	0.0074 (9)
C21	0.0185 (9)	0.0210 (10)	0.0213 (10)	−0.0038 (8)	−0.0049 (8)	0.0092 (8)
C22	0.0278 (11)	0.0189 (10)	0.0224 (11)	−0.0020 (8)	−0.0037 (9)	0.0072 (8)
C23	0.0363 (13)	0.0244 (11)	0.0229 (11)	−0.0036 (9)	−0.0093 (9)	0.0075 (9)
C24	0.0278 (12)	0.0304 (12)	0.0351 (13)	−0.0037 (9)	−0.0114 (10)	0.0180 (10)
C25	0.0189 (10)	0.0329 (12)	0.0388 (14)	−0.0009 (9)	−0.0020 (9)	0.0169 (11)
C26	0.0208 (10)	0.0285 (11)	0.0241 (11)	−0.0017 (8)	−0.0003 (8)	0.0106 (9)
C27	0.0356 (13)	0.0276 (12)	0.0238 (12)	0.0036 (10)	−0.0008 (10)	−0.0007 (9)
C31	0.0240 (10)	0.0191 (10)	0.0246 (11)	−0.0047 (8)	−0.0036 (8)	0.0071 (8)
C32	0.0263 (11)	0.0250 (11)	0.0458 (15)	−0.0028 (9)	−0.0054 (10)	0.0163 (11)
C33	0.0431 (15)	0.0371 (14)	0.0512 (17)	−0.0047 (12)	−0.0136 (13)	0.0285 (13)
C34	0.0532 (17)	0.0430 (15)	0.0327 (14)	−0.0103 (13)	−0.0079 (12)	0.0223 (12)
C35	0.0398 (14)	0.0318 (13)	0.0254 (12)	−0.0061 (10)	0.0022 (10)	0.0079 (10)
C36	0.0282 (11)	0.0220 (10)	0.0240 (11)	−0.0017 (9)	0.0001 (9)	0.0071 (9)
C37	0.0345 (14)	0.0390 (15)	0.068 (2)	0.0120 (12)	−0.0004 (14)	0.0223 (15)
C41	0.0141 (9)	0.0180 (9)	0.0214 (10)	−0.0003 (7)	−0.0011 (7)	0.0060 (8)
C42	0.0131 (9)	0.0187 (9)	0.0322 (11)	0.0017 (7)	0.0032 (8)	0.0122 (9)
C43	0.0162 (9)	0.0292 (11)	0.0352 (12)	0.0002 (8)	−0.0047 (9)	0.0183 (10)
C44	0.0253 (11)	0.0277 (11)	0.0224 (11)	−0.0044 (9)	−0.0063 (8)	0.0106 (9)
C45	0.0249 (10)	0.0191 (10)	0.0234 (11)	0.0018 (8)	0.0006 (8)	0.0065 (8)
C46	0.0156 (9)	0.0186 (9)	0.0250 (11)	0.0031 (7)	0.0002 (8)	0.0070 (8)
C47	0.0182 (10)	0.0231 (11)	0.0458 (14)	0.0075 (8)	0.0056 (9)	0.0119 (10)
C51	0.0227 (11)	0.0487 (16)	0.0276 (13)	0.0018 (11)	−0.0018 (10)	−0.0055 (11)
C52	0.0322 (13)	0.0395 (14)	0.0318 (13)	0.0008 (11)	−0.0006 (10)	0.0034 (11)
C53	0.0492 (17)	0.0384 (15)	0.0428 (16)	0.0208 (13)	−0.0235 (13)	−0.0173 (13)
C54	0.0509 (17)	0.0410 (16)	0.0393 (16)	0.0132 (13)	−0.0076 (13)	−0.0037 (13)
C55	0.078 (2)	0.0315 (14)	0.0369 (16)	0.0026 (14)	−0.0021 (15)	0.0048 (12)
C56	0.067 (2)	0.0321 (14)	0.0262 (13)	0.0147 (13)	0.0057 (13)	0.0003 (11)
C57	0.065 (2)	0.0374 (16)	0.0441 (17)	−0.0048 (14)	−0.0032 (15)	0.0111 (13)
C61	0.0137 (9)	0.0188 (9)	0.0228 (10)	0.0021 (7)	0.0041 (8)	0.0055 (8)
C62	0.0202 (10)	0.0255 (11)	0.0238 (11)	−0.0016 (8)	−0.0006 (8)	0.0076 (9)
C63	0.0217 (10)	0.0247 (11)	0.0285 (12)	−0.0024 (8)	−0.0019 (9)	0.0060 (9)
C64	0.0210 (10)	0.0229 (10)	0.0337 (12)	0.0038 (8)	0.0052 (9)	0.0105 (9)
C65	0.0211 (10)	0.0275 (11)	0.0283 (11)	0.0070 (8)	0.0032 (9)	0.0136 (9)
C66	0.0155 (9)	0.0255 (10)	0.0219 (10)	0.0038 (8)	0.0018 (8)	0.0060 (8)
C67	0.0363 (14)	0.0483 (16)	0.0307 (13)	−0.0197 (12)	−0.0154 (11)	0.0199 (12)

### Geometric parameters (Å, º)

**Table:**

Pd—P1	2.2254 (9)	C34—C35	1.385 (3)
Pd—P2	2.2296 (9)	C34—H34	0.9500
Pd—Cl1	2.3375 (9)	C35—C36	1.389 (3)
Pd—Cl2	2.3164 (9)	C35—H35	0.9500
P1—O1	1.5784 (16)	C36—H36	0.9500
P1—O2	1.5868 (16)	C37—H37A	0.9800
P1—O3	1.5905 (16)	C37—H37B	0.9800
P2—O4	1.5813 (16)	C37—H37C	0.9800
P2—O5	1.5894 (16)	C41—C46	1.385 (3)
P2—O6	1.5946 (16)	C41—C42	1.394 (3)
O1—C11	1.411 (3)	C42—C43	1.390 (3)
O2—C21	1.418 (3)	C42—C47	1.504 (3)
O3—C31	1.408 (3)	C43—C44	1.386 (3)
O4—C41	1.410 (3)	C43—H43	0.9500
O5—C51	1.456 (3)	C44—C45	1.387 (3)
O6—C61	1.424 (3)	C44—H44	0.9500
C11—C16	1.389 (3)	C45—C46	1.387 (3)
C11—C12	1.390 (3)	C45—H45	0.9500
C12—C13	1.403 (3)	C46—H46	0.9500
C12—C17	1.504 (3)	C47—H47A	0.9800
C13—C14	1.385 (3)	C47—H47B	0.9800
C13—H13	0.9500	C47—H47C	0.9800
C14—C15	1.389 (3)	C51—C52	1.370 (4)
C14—H14	0.9500	C51—C56	1.425 (4)
C15—C16	1.384 (3)	C52—C53	1.394 (4)
C15—H15	0.9500	C52—C57	1.459 (4)
C16—H16	0.9500	C53—C54	1.386 (4)
C17—H17A	0.9800	C53—H53	0.9500
C17—H17B	0.9800	C54—C55	1.382 (4)
C17—H17C	0.9800	C54—H54	0.9500
C21—C26	1.382 (3)	C55—C56	1.392 (4)
C21—C22	1.389 (3)	C55—H55	0.9500
C22—C23	1.393 (3)	C56—H56	0.9500
C22—C27	1.508 (3)	C57—H57A	0.9800
C23—C24	1.391 (3)	C57—H57B	0.9800
C23—H23	0.9500	C57—H57C	0.9800
C24—C25	1.383 (3)	C61—C66	1.384 (3)
C24—H24	0.9500	C61—C62	1.391 (3)
C25—C26	1.393 (3)	C62—C63	1.397 (3)
C25—H25	0.9500	C62—C67	1.501 (3)
C26—H26	0.9500	C63—C64	1.385 (3)
C27—H27A	0.9800	C63—H63	0.9500
C27—H27B	0.9800	C64—C65	1.385 (3)
C27—H27C	0.9800	C64—H64	0.9500
C31—C36	1.382 (3)	C65—C66	1.390 (3)
C31—C32	1.395 (3)	C65—H65	0.9500
C32—C33	1.391 (3)	C66—H66	0.9500
C32—C37	1.500 (3)	C67—H67A	0.9800
C33—C34	1.376 (3)	C67—H67B	0.9800
C33—H33	0.9500	C67—H67C	0.9800
			
Cl1—Pd—Cl2	90.59 (2)	C34—C35—H35	120.1
P1—Pd—P2	94.07 (2)	C36—C35—H35	120.1
P1—Pd—Cl1	90.35 (2)	C31—C36—C35	118.9 (2)
P2—Pd—Cl2	85.09 (2)	C31—C36—H36	120.5
P2—Pd—Cl1	175.50 (2)	C35—C36—H36	120.5
O1—P1—O2	105.65 (9)	C32—C37—H37A	109.5
O1—P1—O3	98.71 (9)	C32—C37—H37B	109.5
O2—P1—O3	104.71 (9)	H37A—C37—H37B	109.5
O1—P1—Pd	116.84 (7)	C32—C37—H37C	109.5
O2—P1—Pd	109.27 (7)	H37A—C37—H37C	109.5
O3—P1—Pd	120.08 (7)	H37B—C37—H37C	109.5
O4—P2—O5	100.99 (9)	C46—C41—C42	123.0 (2)
O4—P2—O6	105.81 (9)	C46—C41—O4	121.3 (2)
O5—P2—O6	102.97 (9)	C42—C41—O4	115.7 (2)
O4—P2—Pd	117.48 (7)	C43—C42—C41	116.5 (2)
O5—P2—Pd	114.98 (7)	C43—C42—C47	122.0 (2)
O6—P2—Pd	112.92 (7)	C41—C42—C47	121.5 (2)
C11—O1—P1	125.1 (2)	C44—C43—C42	121.8 (2)
C21—O2—P1	127.9 (2)	C44—C43—H43	119.1
C31—O3—P1	126.6 (2)	C42—C43—H43	119.1
C41—O4—P2	126.6 (2)	C43—C44—C45	120.1 (2)
C51—O5—P2	127.9 (2)	C43—C44—H44	119.9
C61—O6—P2	122.4 (2)	C45—C44—H44	119.9
C16—C11—C12	123.0 (2)	C46—C45—C44	119.7 (2)
C16—C11—O1	120.7 (2)	C46—C45—H45	120.2
C12—C11—O1	116.2 (2)	C44—C45—H45	120.2
C11—C12—C13	116.1 (2)	C41—C46—C45	118.9 (2)
C11—C12—C17	122.0 (2)	C41—C46—H46	120.6
C13—C12—C17	121.9 (2)	C45—C46—H46	120.6
C14—C13—C12	122.1 (2)	C42—C47—H47A	109.5
C14—C13—H13	119.0	C42—C47—H47B	109.5
C12—C13—H13	119.0	H47A—C47—H47B	109.5
C13—C14—C15	119.8 (2)	C42—C47—H47C	109.5
C13—C14—H14	120.1	H47A—C47—H47C	109.5
C15—C14—H14	120.1	H47B—C47—H47C	109.5
C16—C15—C14	119.8 (2)	C52—C51—C56	124.4 (3)
C16—C15—H15	120.1	C52—C51—O5	118.3 (3)
C14—C15—H15	120.1	C56—C51—O5	117.1 (3)
C15—C16—C11	119.1 (2)	C51—C52—C53	116.6 (3)
C15—C16—H16	120.4	C51—C52—C57	120.4 (3)
C11—C16—H16	120.4	C53—C52—C57	123.0 (3)
C12—C17—H17A	109.5	C54—C53—C52	121.6 (3)
C12—C17—H17B	109.5	C54—C53—H53	119.2
H17A—C17—H17B	109.5	C52—C53—H53	119.2
C12—C17—H17C	109.5	C55—C54—C53	120.1 (3)
H17A—C17—H17C	109.5	C55—C54—H54	120.0
H17B—C17—H17C	109.5	C53—C54—H54	120.0
C26—C21—C22	123.4 (2)	C54—C55—C56	121.3 (3)
C26—C21—O2	120.7 (2)	C54—C55—H55	119.3
C22—C21—O2	115.8 (2)	C56—C55—H55	119.3
C21—C22—C23	116.7 (2)	C55—C56—C51	115.9 (3)
C21—C22—C27	121.0 (2)	C55—C56—H56	122.0
C23—C22—C27	122.3 (2)	C51—C56—H56	122.0
C24—C23—C22	121.4 (2)	C52—C57—H57A	109.5
C24—C23—H23	119.3	C52—C57—H57B	109.5
C22—C23—H23	119.3	H57A—C57—H57B	109.5
C25—C24—C23	120.0 (2)	C52—C57—H57C	109.5
C25—C24—H24	120.0	H57A—C57—H57C	109.5
C23—C24—H24	120.0	H57B—C57—H57C	109.5
C24—C25—C26	120.2 (2)	C66—C61—C62	122.8 (2)
C24—C25—H25	119.9	C66—C61—O6	119.1 (2)
C26—C25—H25	119.9	C62—C61—O6	118.0 (2)
C21—C26—C25	118.3 (2)	C61—C62—C63	116.8 (2)
C21—C26—H26	120.8	C61—C62—C67	121.9 (2)
C25—C26—H26	120.8	C63—C62—C67	121.2 (2)
C22—C27—H27A	109.5	C64—C63—C62	121.7 (2)
C22—C27—H27B	109.5	C64—C63—H63	119.2
H27A—C27—H27B	109.5	C62—C63—H63	119.2
C22—C27—H27C	109.5	C63—C64—C65	119.8 (2)
H27A—C27—H27C	109.5	C63—C64—H64	120.1
H27B—C27—H27C	109.5	C65—C64—H64	120.1
C36—C31—C32	122.7 (2)	C64—C65—C66	120.2 (2)
C36—C31—O3	121.9 (2)	C64—C65—H65	119.9
C32—C31—O3	115.3 (2)	C66—C65—H65	119.9
C33—C32—C31	116.4 (2)	C61—C66—C65	118.7 (2)
C33—C32—C37	122.2 (2)	C61—C66—H66	120.6
C31—C32—C37	121.3 (2)	C65—C66—H66	120.6
C34—C33—C32	122.2 (2)	C62—C67—H67A	109.5
C34—C33—H33	118.9	C62—C67—H67B	109.5
C32—C33—H33	118.9	H67A—C67—H67B	109.5
C33—C34—C35	120.0 (2)	C62—C67—H67C	109.5
C33—C34—H34	120.0	H67A—C67—H67C	109.5
C35—C34—H34	120.0	H67B—C67—H67C	109.5
C34—C35—C36	119.7 (2)		
			
P2—Pd—P1—O1	70.82 (8)	O2—C21—C26—C25	−177.6 (3)
Cl1—Pd—P1—O1	−108.35 (8)	C24—C25—C26—C21	0.3 (3)
P2—Pd—P1—O2	−49.00 (8)	P1—O3—C31—C36	−37.3 (2)
Cl1—Pd—P1—O2	131.83 (8)	P1—O3—C31—C32	144.8 (2)
P2—Pd—P1—O3	−169.89 (8)	C36—C31—C32—C33	−0.3 (3)
Cl1—Pd—P1—O3	10.94 (8)	O3—C31—C32—C33	177.6 (3)
P1—Pd—P2—O4	−148.52 (8)	C36—C31—C32—C37	−178.9 (3)
Cl2—Pd—P2—O4	36.76 (8)	O3—C31—C32—C37	−0.9 (3)
P1—Pd—P2—O5	92.84 (8)	C31—C32—C33—C34	0.4 (3)
Cl2—Pd—P2—O5	−81.88 (8)	C37—C32—C33—C34	178.9 (3)
P1—Pd—P2—O6	−24.92 (8)	C32—C33—C34—C35	−0.3 (3)
Cl2—Pd—P2—O6	160.36 (8)	C33—C34—C35—C36	0.0 (3)
O2—P1—O1—C11	143.8 (2)	C32—C31—C36—C35	0.1 (3)
O3—P1—O1—C11	−108.2 (2)	O3—C31—C36—C35	−177.7 (3)
Pd—P1—O1—C11	22.1 (2)	C34—C35—C36—C31	0.1 (3)
O1—P1—O2—C21	71.8 (2)	P2—O4—C41—C46	38.1 (2)
O3—P1—O2—C21	−31.8 (2)	P2—O4—C41—C42	−144.2 (2)
Pd—P1—O2—C21	−161.7 (2)	C46—C41—C42—C43	0.8 (3)
O1—P1—O3—C31	−155.4 (2)	O4—C41—C42—C43	−176.8 (3)
O2—P1—O3—C31	−46.6 (2)	C46—C41—C42—C47	−177.4 (3)
Pd—P1—O3—C31	76.6 (2)	O4—C41—C42—C47	5.0 (3)
O5—P2—O4—C41	165.8 (2)	C41—C42—C43—C44	−1.5 (3)
O6—P2—O4—C41	−87.1 (1)	C47—C42—C43—C44	176.6 (3)
Pd—P2—O4—C41	39.9 (1)	C42—C43—C44—C45	1.0 (3)
O4—P2—O5—C51	−15.2 (2)	C43—C44—C45—C46	0.3 (3)
O6—P2—O5—C51	−124.4 (2)	C42—C41—C46—C45	0.4 (3)
Pd—P2—O5—C51	112.4 (2)	O4—C41—C46—C45	177.9 (3)
O4—P2—O6—C61	−50.1 (2)	C44—C45—C46—C41	−1.0 (3)
O5—P2—O6—C61	55.5 (2)	P2—O5—C51—C52	−110.9 (3)
Pd—P2—O6—C61	−179.9 (2)	P2—O5—C51—C56	74.7 (3)
P1—O1—C11—C16	43.3 (2)	C56—C51—C52—C53	−1.5 (4)
P1—O1—C11—C12	−140.1 (2)	O5—C51—C52—C53	−175.5 (4)
C16—C11—C12—C13	1.1 (3)	C56—C51—C52—C57	178.7 (4)
O1—C11—C12—C13	−175.5 (3)	O5—C51—C52—C57	4.7 (4)
C16—C11—C12—C17	179.6 (3)	C51—C52—C53—C54	−0.4 (4)
O1—C11—C12—C17	3.0 (3)	C57—C52—C53—C54	179.4 (4)
C11—C12—C13—C14	−1.6 (3)	C52—C53—C54—C55	0.3 (4)
C17—C12—C13—C14	179.9 (3)	C53—C54—C55—C56	1.6 (4)
C12—C13—C14—C15	0.7 (3)	C54—C55—C56—C51	−3.2 (4)
C13—C14—C15—C16	0.9 (3)	C52—C51—C56—C55	3.3 (4)
C14—C15—C16—C11	−1.4 (3)	O5—C51—C56—C55	177.3 (4)
C12—C11—C16—C15	0.4 (3)	P2—O6—C61—C66	76.2 (2)
O1—C11—C16—C15	176.8 (3)	P2—O6—C61—C62	−107.8 (2)
P1—O2—C21—C26	−49.2 (2)	C66—C61—C62—C63	−2.1 (3)
P1—O2—C21—C22	134.2 (2)	O6—C61—C62—C63	−178.1 (3)
C26—C21—C22—C23	1.3 (3)	C66—C61—C62—C67	175.0 (3)
O2—C21—C22—C23	177.8 (3)	O6—C61—C62—C67	−0.9 (3)
C26—C21—C22—C27	−178.9 (3)	C61—C62—C63—C64	0.7 (3)
O2—C21—C22—C27	−2.3 (3)	C67—C62—C63—C64	−176.5 (3)
C21—C22—C23—C24	−0.3 (3)	C62—C63—C64—C65	1.0 (3)
C27—C22—C23—C24	179.8 (3)	C63—C64—C65—C66	−1.4 (3)
C22—C23—C24—C25	−0.6 (3)	C62—C61—C66—C65	1.8 (3)
C23—C24—C25—C26	0.6 (3)	O6—C61—C66—C65	177.7 (3)
C22—C21—C26—C25	−1.3 (3)	C64—C65—C66—C61	0.0 (3)

### Hydrogen-bond geometry (Å, º)

**Table:**

*D*—H···*A*	*D*—H	H···*A*	*D*···*A*	*D*—H···*A*
C17—H17*A*···Cl2^i^	0.98	2.72	3.521 (3)	139
C45—H45···Cl1^ii^	0.95	2.91	3.680 (3)	139

Symmetry codes: (i) *x*−1, *y*, *z*; (ii) −*x*+1, −*y*+1, −*z*+1.

### Intermolecular π–π interactions (Å, °).

**Table:**

CgI	CgJ	Cg···Cg	Interplanar distance	Offset
1	1^iii^	3.758 (4)	3.409 (4)	1.582 (4)
2	2^iv^	4.651 (4)	4.164 (4)	2.072 (4)

Cg1 denotes the centroid of ring C11-C16; Cg2 of ring C41-C46. Cg···Cg is the distance between ring centroids. The interplanar distance is the perpendicular distance of CgI from ring J plane. The offset is the lateral displacement of ring I relative to ring J. The planes of the I and J rings are parallel.Symmetry codes: (iii) -x, -y+1, -z+1; (iv) -x+1, -y, -z+1.

## References

[bb1] Aullón, G., Bellamy, D., Brammer, L., Bruton, E. & Orpen, A. G. (1998). *Chem. Commun.* pp. 653–654.

[bb2] Błaszczyk, I., Trzeciak, A. M. & Ziółkowski, J. J. (2009). *Catal. Lett.* **133**, 262–266.

[bb3] Clark, R. C. & Reid, J. S. (1995). *Acta Cryst.* A**51**, 887–897.

[bb4] Cosier, J. & Glazer, A. M. (1986). *J. Appl. Cryst.* **19**, 105–107.

[bb5] Desiraju, G. R. & Steiner, T. (1999). *The Weak Hydrogen Bond in Structural Chemistry and Biology* New York: Oxford University Press Inc.

[bb6] Farrugia, L. J. (1997). *J. Appl. Cryst.* **30**, 565.

[bb7] McGaughey, G. B., Gagné, M. & Rappé, A. K. (1998). *J. Biol. Chem.* **273**, 15458–15463. 10.1074/jbc.273.25.15458 9624131

[bb8] Orpen, A. G., Brammer, L., Allen, F. H., Kennard, O., Watson, D. G. & Taylor, R. (1989). *J. Chem. Soc. Dalton Trans.* pp. S1–83.

[bb9] Oxford Diffraction (2010). *CrysAlis CCD* and *CrysAlis RED* Oxford Diffraction Ltd, Wrocław, Poland.

[bb10] Sabounchei, S. J., Naghipour, A. & Bickley, J. F. (2000). *Acta Cryst.* C**56**, e280.

[bb11] Sheldrick, G. M. (2008). *Acta Cryst.* A**64**, 112–122. 10.1107/S0108767307043930 18156677

[bb12] Sonogashira, K., Tohda, Y. & Nagihara, N. (1975). *Tetrahedron Lett.* **16**, 4467–4470.

[bb13] Trzeciak, A. M., Bartosz-Bechowski, H., Ciunik, Z., Niesyty, K. & Ziółkowski, J. J. (2001). *Can. J. Chem.* **79**, 752–759.

